# Active Barrier Coating for Packaging Paper with Controlled Release of Sunflower Oils

**DOI:** 10.3390/molecules26123561

**Published:** 2021-06-10

**Authors:** Pieter Samyn

**Affiliations:** Analytical and Circular Chemistry, Hasselt University, 3590 Diepenbeek, Belgium; pieter.samyn@outlook.be

**Keywords:** paper, packaging, coating, barrier, encapsulation, release, vegetable oil, hydrophobicity, oleophobicity

## Abstract

The use of paper as a sustainable packaging material is favored, but it lacks sufficient barrier properties in terms of water repellence and oil resistance. Novel approaches consider active packaging materials or coatings with controlled release providing additional functionality for delivery of specific components to the surface. In this study, the development of a waterborne coating with organic nanoparticles and encapsulated sunflower oils is presented as a system for thermal release of the oil and on-demand tuning of the final barrier properties of the paper substrate. After synthesis of the nanoparticles, it seems that the encapsulation of various grades of sunflower oil (i.e., either poly-unsaturated or mono-unsaturated) strongly affects the encapsulation efficiency and thermal release profiles. The water contact angles are controlled by the oil release and chemical surface composition of the coating upon thermal heating. The oil resistance of the paper improves as a more continuous oil film is formed during thermal release. In particular, the chemical surface composition of the paper coatings is detailed by means of micro-Raman spectroscopy and surface imaging, which provide an analytical quantification tool to evaluate surface coverage, oil delivery, and variations in organic coating moieties.

## 1. Introduction

Today, the packaging industry has urgency for more sustainable solutions and new packaging concepts [1], combining both a rational selection of materials and the introduction of additional functionalities. The improvement of quality, safety, and shelf lifetime of packaged goods is important for fresh food applications [2], while the protection against ingress of contaminants, such as moisture or oil, is also important in dry goods [3]. Whereas packaging coatings were historically seen as an inert barrier layer [4], novel research into active packaging coatings considers the interaction with the environment in order to optimize preservation [5,6]. Controlled release packaging (CRP) has been introduced as a novel generation of packaging materials [7,8,9], which provide active release of compounds over time or depending on their environment at desirable rates. In parallel, the consumption of traditional fossil-based polymers needs to be reduced, and a transition towards more naturally-based materials such as papers and bio-renewable polymers is necessary [10,11].

A primary concern for paper-based packaging is to improve its barrier properties; in particular, the water repellence needs to be controlled [12]. The hygroscopic properties and swelling are caused by the presence of hydroxyl groups and the hydrophilic nature of cellulose fibers, which therefore deteriorate water uptake and oxygen barrier properties under high humidity. The protection of paper-based packaging is ensured through additional coating layers and is often done industrially through lamination with a polymer [13]. However, the lamination reduces the paper recyclability, and alternative methods based on hydrophobic dispersion coatings have been developed [14,15,16]. In most cases, the hydrophobicity of cellulose is introduced by direct chemical modification through grafting [17,18], or with coating deposition of silylated soybean oil [19] or silicone oil [20]. The modification with vegetable oil triglycerides offers a bio-based alternative, unless they are provided in aqueous emulsions [21]. However, the chemical surface modification or coating of the substrate mostly offers a “passive” layer with permanent properties. The more “active” methods for control of surface hydrophobicity employ in situ formation of self-organized structures [22], application of an external trigger (e.g., light or temperature) to tune the properties on demand [23,24], or introduction of phase-change materials [25,26]. The latter methods have the advantage that synthesis conditions of a coating can be decoupled from the hydrophobicity requirement, which is finally regulated after deposition.

The encapsulation of various compounds in specialty packaging (e.g., anti-static, anti-fungal, baking paper) and stimuli-responsive materials is highly efficient to introduce additional functionalities [27,28], and may include bio-active ingredients [29], antimicrobials [30], anti-oxidants [31], light blockers [32], and hydrophobic moieties or essential oils [33]. The micro-encapsulation processes of oil were primarily developed for antimicrobial paper coatings [34]. Such microcapsules could be prepared through freeze-drying and conservation in combination with supercritical fluids [35]. More recently, the advanced encapsulation technologies for anti-microbial components were developed in parallel with nanotechnological achievements, such as formulations of nano-emulsions, core-shell nanofibers, cyclodextrines and liposomes [36]. The nanoencapsulation of oils is a promising alternative to improve the bioavailability of encapsulated matter [37]: in particular, close control of the synthesis conditions and use of different oils allow one to change the morphological parameters such as size and polydispersity. The passive nanoparticles with solid shell may provide on-demand release through mechanical bursting, but tuning of the nanoparticles’ walls towards a more porous structure is more efficient for controlled release mechanisms under changes in temperature [38]. The encapsulation of hydrophobic agents in packaging coatings was done for, for example, loading of hydrophobic aroma [39], or dispersion of carbon black during miniemulsion polymerization of a styrene/acrylate coating [40]. As such, bioavailability of hydrophobic bioactive compounds such as phenolics, oils, and fatty acids can be enhanced when they are protected within nanocapsules [41], not only for application in the food industry but also when supplied through the packaging material. The encapsulation of hydrophobic food flavors into nanocarriers also enhances the thermal stability and oxidation resistance of the oil [42]. In particular, the encapsulation of oils onto cardboard has extended the shelf life of fruits and vegetables, as it highly decreases the evaporation of the oils while guaranteeing a controlled release [43,44]. The inclusion of fragrances as microcapsules in UV-curable coatings for wrapping paper improved the printability and gave burst release by rubbing [45]. The innovative applications for nanoencapsulation are in their earliest development stage, with huge potential as stimuli-responsive materials and functional coatings for food preservation [46,47]. The different stimuli-responsive release mechanisms for coatings include, for example, light- and pH-sensitive materials that provide on-demand self-repairing and superhydrophobic properties [48], or temperature-responsive polymers with controlled release and self-cleaning properties [49].

The active control of hydrophobic barrier properties of papers through a coating with nanoencapsulation and thermal release of oils has not yet been described, and can be introduced as a synergistic effect of encapsulated oils. In this study, it is demonstrated how the selection of oil type influences the release properties and consequently the surface hydrophobicity of coated papers upon selection of a heating temperature as external trigger. In particular, the chemical composition of the paper coatings are detailed by spectroscopic imaging that provides a quantification tool to evaluate the required surface properties.

## 2. Experimental Details

### 2.1. Materials

**Figure 1:**
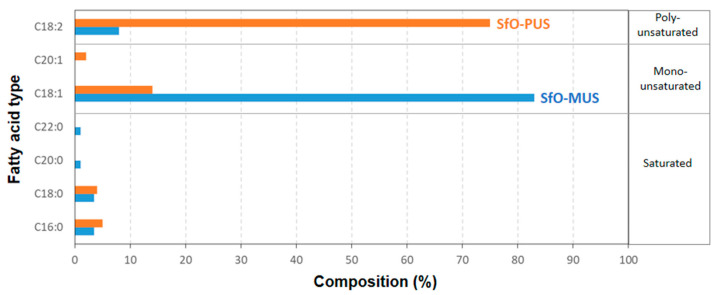
Fatty acid composition of sunflower oil (SfO) used for encapsulation in polymer nanoparticles, for SfO-PUS (poly-unsaturated) and SfO-MUS (mono-unsaturated).

The hybrid polymer nanoparticles were synthesized similarly to a protocol that was reported earlier [50], containing a polymer phase of poly(styrene-co-maleimide) or SMIand an encapsulated oil phase. In particular, two types of sunflower oil (SfO) were provided by Cargill Agricola S/A (Mairinque, SP, Brazil), with different fatty acid compositions (Figure 1). The oil composition was determined by gas chromatography after derivatization into methyl esters following the AOCS (American Oil Chemists Society) standard method Ce 1-62. The fatty acids are indicated as Cx:y components, with x the number of carbon atoms and y the number of double bonds. The first type of sunflower oil, SfO-PUS, has a high concentration of poly-unsaturated fatty acids, that is, C18:2 (linoleic acid), while the second type of sunflower oil, SfO-MUS, has a high concentration of mono-unsaturated fatty acids, that is C18:1 (oleic acid). In parallel, the iodine values (I.V.) of the sunflower oils were determined by standardized titration methods (ASTM D5554-15 Standard test method for determination of the iodine value of fats and oils) and are significantly different, that is, I.V. = 125 (SfO-PUS) and I.V. = 93 (SfO-MUS). The oils were separately added into an aqueous reaction mixture together with the poly(styrene-co-maleic anhydride) or SMA precursor (26 mol-% maleic anhydride, 74 mol-% styrene) provided by Polyscope (Geleen, The Netherlands) and imidized in the presence of ammonium hydroxide. The nanoparticles SMI/SfO-PUS and SMI/SfO-MUS were synthesized with 50% (*w*/*w*) oil relative to the polymer. Thus, the imidization reaction was performed in an autoclave reactor of 1 L, while adding an amount of 212 g styrene maleic anhydride, 212 g oil, 38.7 g NH_4_OH, and 380 g water. The reaction temperature was gradually augmented to 160 °C over about 4 h. As a result, a stable milky-white colored waterborne dispersion was obtained without phase separation between the organic and the oil phase at pH = 5.5 to 5.7, solid content of 50%, and viscosity of 130 to 140 cp.

### 2.2. Coating Application

As a substrate for coating application, a kraft pulp paper grade of a 100 g/m^2^ grammage with internal starch sizing and calendared base top coating was used (Mondi, Addlestone, UK). The aqueous nanoparticle suspensions of SMI/SfO-PUS and SMI/SfO-MUS were applied through a lab-scale bar coater (K303 Multicoater, RK Print Coat Instruments Ltd., Litlington, Royston, UK) until a dry coat thickness of about 5 µm was obtained corresponding to a dry coat weight of 6.0 ± 0.2 g/m^2^. A green metering bar with close wound wire diameter 0.30 mm was moving at an automated speed of 6 mm/s. The coated papers were subsequently placed in a circulating hot-air oven at 120 °C for 2 min in order to accelerate the evaporation of water into a solid film. The sheets were further conditioned under controlled laboratory atmosphere (23 °C, 60% RH) during one week for full drying.

The thermal release of oil from the paper coatings was induced during heating of coated paper pieces of 5 × 5 cm^2^, placing them horizontally and coating side up in a pre-heated hot-air oven, for a given time of 6 h at different temperatures of 125, 135, 150, 180, 220, and 250 °C. The temperatures were selected over a broad range to cover a screening study including different temperature intervals that cover variations in chemical reactivity of the SMI polymer phase and the glass transition temperature (*T_g_*) of the nanoparticles. The selected time allows for complete homogenization and stabilization of the oil release under steady-state conditions. The coating samples were unloaded from the oven and cooled under ambient air conditions, further resting for one week before further surface characterization.

### 2.3. Characterization Methods

Micro-Raman spectroscopy of coated papers was captured on the top surfaces, using a dispersive Raman Flex 400 spectrometer (Perkin Elmer, Rodgau, Germany) with a coupled optical fiber into an optical light microscope BX 51 (Olympus, Hamburg, Germany). The emitted laser wavelength of 785 nm was generated through a laser diode in the micro-Raman module 300 (Perkin Elmer, Rodgau, Germany) with an intensity that was reduced towards 40 mW near the sample position. The single-point Raman spectra were collected in a range of 600–3200 cm^−1^ with a resolution of 4 cm^−1^ and averaged over 10 scans per position. An exposure time of 25 s per position was selected as optimum condition for a good signal-to-noise ratio. The chemical surface mapping was done over a surface area of 1 × 1 mm^2^, where the spectra were interpolated between 20 × 20 single-point measurements with intermediate point distance of 0.05 mm. The data was further processed by Surface Image software (version R1.7.0.04), plotting the band ratio maps, single wavenumber maps, and chemical surface maps as visual surface plots. In addition, Image J 1.52a software (version 1.8.0_112) was used for processing of the surface maps. The surface area coverage was determined by selection of an appropriate threshold value that represented the features related to the oil deposits. The greyscale values were determined by transformation into an 8-bit image and reading of the mean value from the greyscale histogram.

The surface morphology was evaluated with scanning electron microscopy (SEM) using a desktop TM3000 instrument (Hitachi, Krefeld, Germany) at magnifications of 800×, 2000×, and 20,000×. The samples were evaluated without need for a gold sputter layer, under application of a constant acceleration voltage of 15 kV (tungsten filament) and working distance of 8.5 mm. The images represent compositional contrast corresponding to the BSE signal. The original nanoparticle morphologies were analyzed by transmission electron microscopy (TEM) on a Leo 912 Omega (Zeiss, Jena, Germany). The nanoparticle suspension was deposited by dipping of a carbon-grid and drying under a nitrogen air flow before examination.

The static water contact angles were measured on an automatic Digidrop goniometer (GBX, Bourg-de-Peage, France), depositing water droplets of 2 µL and fitting the geometry with a predefined tangent procedure to determine an average of left and right contact angles. The surface porosity for coated papers was sufficiently reduced to obtain stable water droplets over a time of at least 15 s. The values were consistently read 2 s after deposition of the droplet. The values are reported as an average of ten measurements, depending on the statistical variation to be within ±2°.

The oil-resistant properties were determined by contact angle measurements with castor oil as a standard probe liquid to verify the wettability of oil. Next, the evaluation according to Tappi 559, known as the KIT oil and grease resistance test, was performed with a mixture series of castor oil, toluene, and n-heptane in different ratios. The testing liquid was placed on the coating and wiped off after 15 s, examining for darkening of the test area. The higher the number of KIT solution that does not consequently cause failure, the better the oil resistance of the sample.

## 3. Test Results

### 3.1. Microscopic Morphology of Paper Coatings

**Figure 2:**
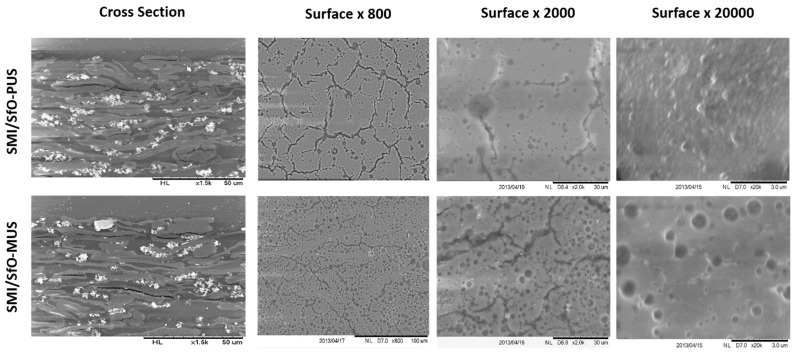
Scanning electron microscopy (SEM) images of original SMI/SfO-PUS and SMI/SfO-MUS coatings on paper substrates, including cross section (left column) and surface view at different magnifications.

The morphology of SMI/SfO-PUS and SMI/SfO-MUS coatings on paper is evaluated by SEM images in Figure 2. The cross sections confirm that the coatings are positioned on top of the paper substrates, with a coating thickness of about 5 µm. The coating indeed covers the paper substrate with a relatively flat top surface, not following the undulations and unevenness of the paper substrate, as a characteristic for the bar coating process. A relatively open structure of the paper and the coating is observed, as the coated papers were intentionally not further calendared and/or no additional binder was added in order to study the original coating morphology. The top surface images show some coating cracks, owing to the drying of aqueous suspensions, together with the presence of separate spots representing the free oil deposits. The cracking of the coating is typically induced by drying phenomena in waterborne dispersions [51]. At higher magnification, the free oil was observed as relatively large encapsulated droplets and was mainly present for the coatings of SMI/SfO-MUS. The free oil droplets are less frequently present at the surface of SMI/SfO-PUS coatings, which show a well-developed surface texture with submicron nanoparticles and encapsulated oil. Although the same concentrations of encapsulated oil and reaction conditions were applied, it reveals that the coating morphology for both coating types with different oils is fairly different. In particular, the small differences in original oil composition, related to the degree of saturation may induce different coating morphologies and accumulation of free oil.

**Figure 3:**
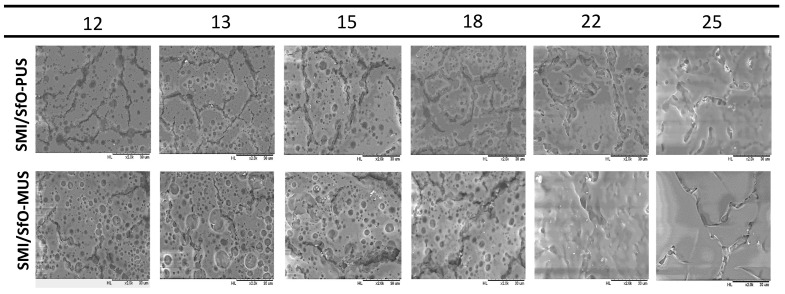
Surface morphology of SMI/SfO-PUS and SMI/SfO-MUS coatings after thermal release of oil at different temperatures (magnification × 2000).

The variations in surface morphology upon thermal heating and the release of oil at different temperatures are represented for both coatings in Figure 3. The surface observations confirm differences in oil exposure of SMI/SfO-MUS and SMI/SfO-PUS coatings with a constantly higher amount of rather large free oil droplets for the SMI/SfO-MUS coatings. The morphology at low temperatures up to 125 °C corresponds well to the original coatings. At higher temperatures of 135–150 °C, the amount of free oil increases with agglomeration of the free oil into some local droplets. At the highest temperatures of 180–250 °C, the additional plastification and smoothening of the coating layer is observed in parallel with surface coverage of oil. The variations in coating morphology can be understood in parallel with previous measurements of the glass transition temperatures *T_g_* of the SMI/SfO-PUS and SMI/SfO-MUS nanoparticles at 150–155 °C [50]. The local flow of the coating above *T_g_* can be attributed to a gradual thermal weakening of the SMI polymer phase and progressive release of the oil.

**Figure 4:**
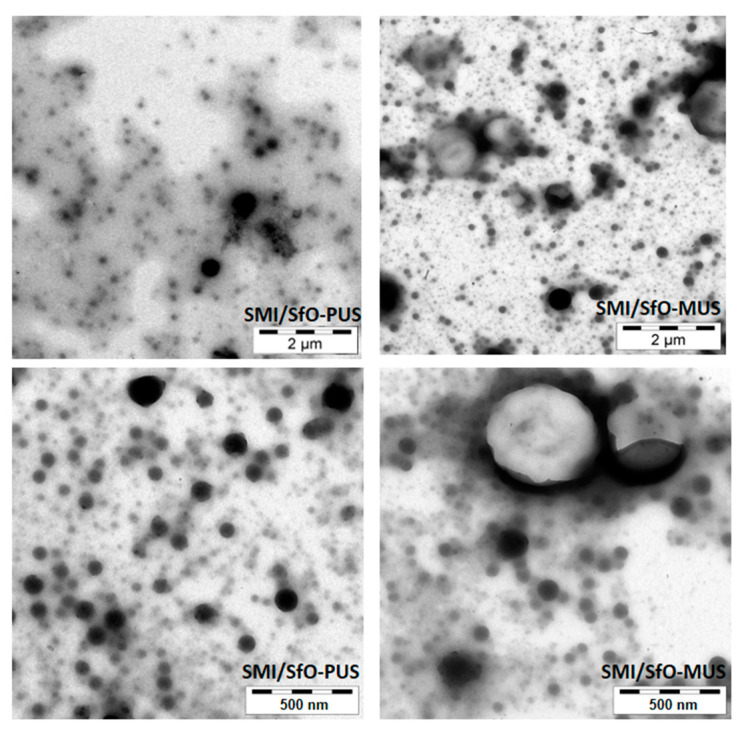
Transmission electron microscopy (TEM) of SMI/SfO-PUS and SMI/SfO-PUS nanoparticles at different magnifications.

The intrinsic morphologies of the SMI/SfO-PUS and SMI/SfO-MUS nanoparticles are evaluated by TEM images in Figure 4, including two levels of magnifications. The dia-meter of the spherical nanoparticles ranges between 20 and 50 nm for both oil types, while the occurrence of larger droplets in the case of SMI/SfO-MUS represents the presence of free oil. The amount of free oil is quantified by spectroscopy in following paragraphs, but it can be revealed that some free oil is situated at the perimeter of the nanoparticles themselves in the case of SMI/SfO-PUS, while the oil also forms a separate phase as large droplets in the case of SMI/SfO-MUS. The presence of some fee oil, therefore, has some binding effect between the nanoparticles during the formation of a coating.

### 3.2. Quantification of Release Profiles from Single-Point Micro-Raman Spectroscopy

**Figure 5:**
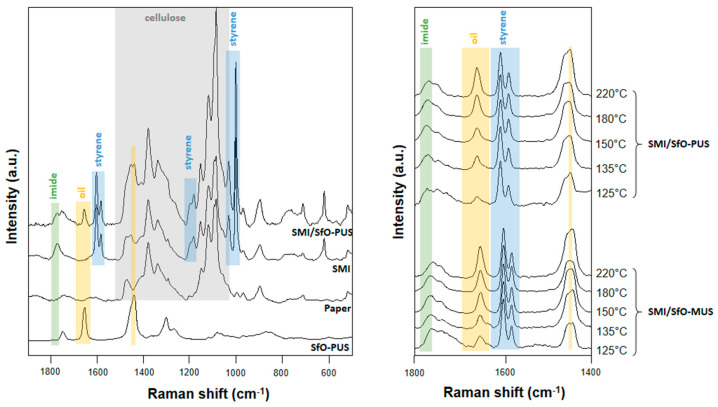
Single-point Raman spectra for SMI/SfO-PUS and SMI/SfO-MUS coatings together with reference materials, and after thermal release of oil at different temperatures.

The single-point measurements of dispersive Raman spectra for coated papers are presented in Figure 5, including comparative measurements for the reference materials (i.e., SfO-PUS oil, uncoated paper, SMI coating without SfO, and SMI/SfO-PUS coating). A detail of the fingerprint region of SMI/SfO-PUS and SMI/SfO-MUS coatings is included, with an evolution of the spectra over different temperatures. All spectra were baseline corrected and normalized over the integrated peak area of the styrene region 1600–1583 cm^−1^ as a chemical non-reactive component. The aromatic structure of styrene did not participate in the chemical reaction for imidization and oil encapsulation, but its structure is thermally stable and did not chemically transform or degrade during the thermal release experiments. The main peaks that are used for relative quantification of the different components of the coated papers are indicated on the spectra. The paper substrate is characterized by typical Raman bands corresponding with cellulose in the region 1500 to 1200 cm^−1^ (H–C–C, H–C–O, C–H–C); 1336 cm^−1^ (CH_2_); and 1170 to 1050 cm^−1^ (C–O, C–C, C–O–C). The SMI polymer phase in the nanoparticles is characterized by the Raman bands at 1780 cm^−1^ (C=O imide I); 1600 to 1583 cm^−1^ (styrene, aromatic); 1182 to 1190 cm^−1^ (styrene C=C); 1032 to 1000 cm^−1^ (C–H styrene); and 900 to 750 cm^−1^ (styrene). The oil phase is particularly represented by the Raman bands at 1748 cm^−1^ (C=O ester); 1657 cm^−1^ (C=C cis); 1442 cm^−1^ (CH2); 1305 cm^−1^ (CH2); and 1266 cm^−1^ (=CH). All components in the present coating system are well separated in the fingerprint region 1800 to 1400 cm^−1^, which is chosen with no overlap or interference between other components. For a constant value of intensities in the styrene band region 1600 to 1583 cm^−1^, the oil band 1748 cm^−1^ and imide band 1780 cm^−1^ irregularly increase with higher temperatures.

**Figure 6:**
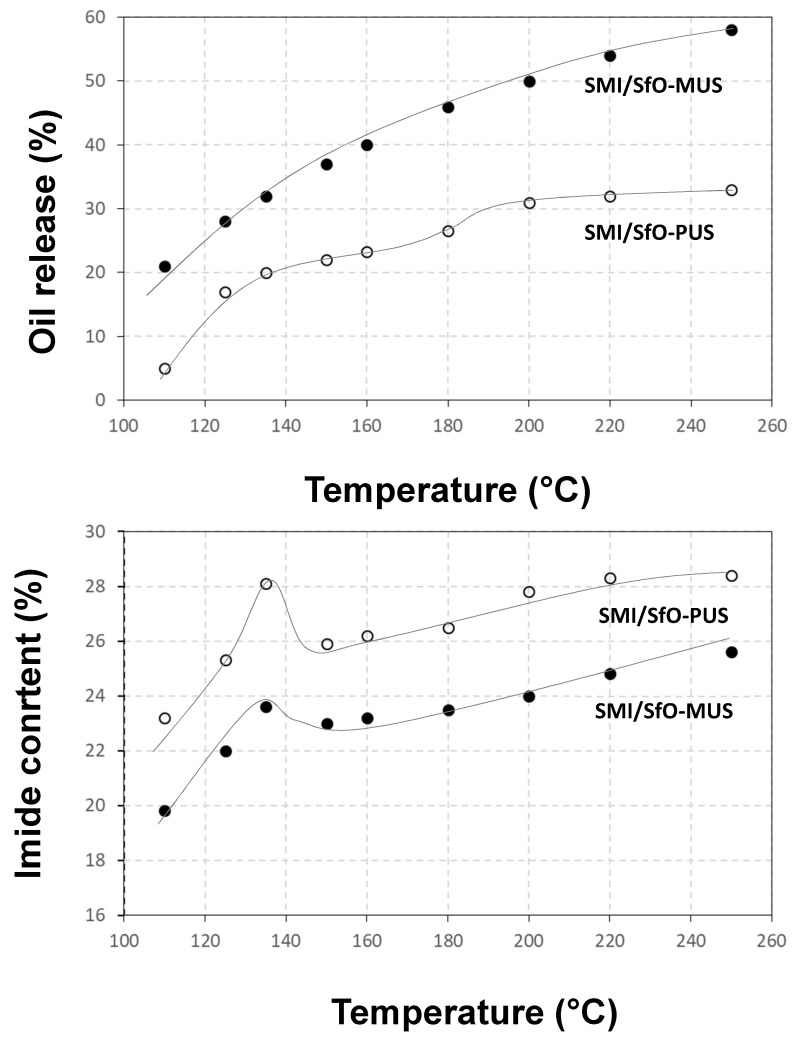
Quantification of chemical characteristics of SMI/SfO-PUS and SMI/SfO-MUS coatings after thermal oil release based on single-point Raman spectra: (**a**) thermal oil release profiles, (**b**) variations in imide content.

The integrated surface areas of those bands are further used for quantification of the oil release and chemical modifications upon heating of the coatings. The calibration of relative Raman intensity ratios at 1748 and 1600 cm^−1^ corresponding to the presence of free oil was confirmed against an external calibration through extraction of the free oil with toluene. The reacted versus free oil content could be determined after calibration with a simple mixture of the oil phase and polymer phase without additional chemical imidization reaction, in a given ratio of 50 wt.-% oil versus styrene maleimide. The calibration takes into account that in the latter case, the unsaturated bonds remain present in the mixed state and are not chemically converted during imidization. The calibration of the relative Raman intensity ratios 1780 to 1600 cm^−1^ (imide/styrene) for determination of imide content was performed with a fully imidized sample, using a styrene maleic anhydride precursor with 26 mol-% maleic anhydride that is fully converted into maleimide. Therefore, the maximum theoretical imide content in the coating material amounts to up to 35 mol-% as calculated from the ratio of imide content (26 mol-%) to styrene content (74 mol-%). The oil exposure and variations in imide content after heating at different temperatures are quantified in Figure 6.

The oil release profile (Figure 6a) can be followed as the amount of free oil in the coating, starting from an original content of 18% for SMI/SfO-MUS and 4% for SMI/SfO-PUS. The free oil content for the original coatings was confirmed through external extraction with toluene, yielding a content of 23% for SMI/SfO-MUS and 7% for SMI/SfO-PUS. From the matching values, it was affirmed that the selected Raman bands provide a suitable quantification method for the thermal release of oil. The exposure of free oil during thermal release follows different trends for both types of encapsulated oil. The oil release is larger for the mono-unsaturated SMI/SfO-MUS compared to the poly-unsaturated SMI/SfO-PUS coatings. The release of mono-unsaturated oil in SMI/SfO-MUS gradually increases with temperature and seems to be little influenced by the surrounding polymer phase. The release of poly-unsaturated oil in SMI/SfO-PUS is retarded and follows a more complex trend that is additionally influenced by transitions in the polymer phase, resulting in either blocking of the release in certain temperature ranges and/or stimulating the release only in a limited temperature range.

The changes in imide content during thermal heating (Figure 6b) follow other trends for both coatings with different types of encapsulated oil. The imide content for SMI/SfO-PUS is significantly higher than for SMI/SfO-MUS coatings, although it follows a similar evolution in terms of temperature, with (i) a first maximum during heating at 135 °C, and (ii) a gradual increase between temperatures of 150 to 250 °C that is somewhat more pronounced for the SMI/SfO-MUS with the lowest imide content. At the highest temperatures, the maximum theoretical imide content of 35 mol-% is not attained, as a residual fraction of ammonolyzed maleic anhydride remains existing in order to regulate the stability of the coating dispersions [50]. The presence of oil clearly interferes with the imidization reaction, as the mono-unsaturated oil MUS is less reactive and hinders the imidization reaction of maleic anhydride to a relatively higher extent, while the more reactive poly-unsaturated oil PUS participates in the imidization reaction and becomes encapsulated to a higher extent.

Although the composition of the SfO types is only slightly different, the imide content is significantly different and influences the thermal release profiles. The nanoparticles with less reactive MUS oil have a lower imide content and consequently experience less interferences between the polymer phase and oil phase. As a result, the amount of free oil is higher and the thermal release during heating is almost not influenced by ongoing imidization. For the nanoparticles with more reactive PUS oil, the imide content is higher and consequently hinders a fluent oil release. The thermal stability and mechanical rigidity of the imide phase might therefore impede the release of oil from its internal structure. In particular, the strong reactivity of the coating upon thermal heating at 135 °C is kinetically favorable for the ongoing imidization reaction [52], corresponding to the stabilization in oil release mechanism. In agreement with previous determination of the glass transition temperature *T_g_* of the nanoparticles, the values of *T_g_* = 156.2 °C for SMI/SfO-PUS and *T_g_* = 152.3 °C for SMI/SfO-PUS reflect the differences in imide content [50]. The relation between thermal oil release and glass transition is most pronounced for SMI/SfO-PUS coatings with high imide content, as the release is enhanced after weakening of the polymer phase. The relation between weakening of the polymer phase and thermal release is less clear for SMI/SfO-PUS coatings with lower imide content, also because the amount of free oil is already relatively high.

**Figure 7:**
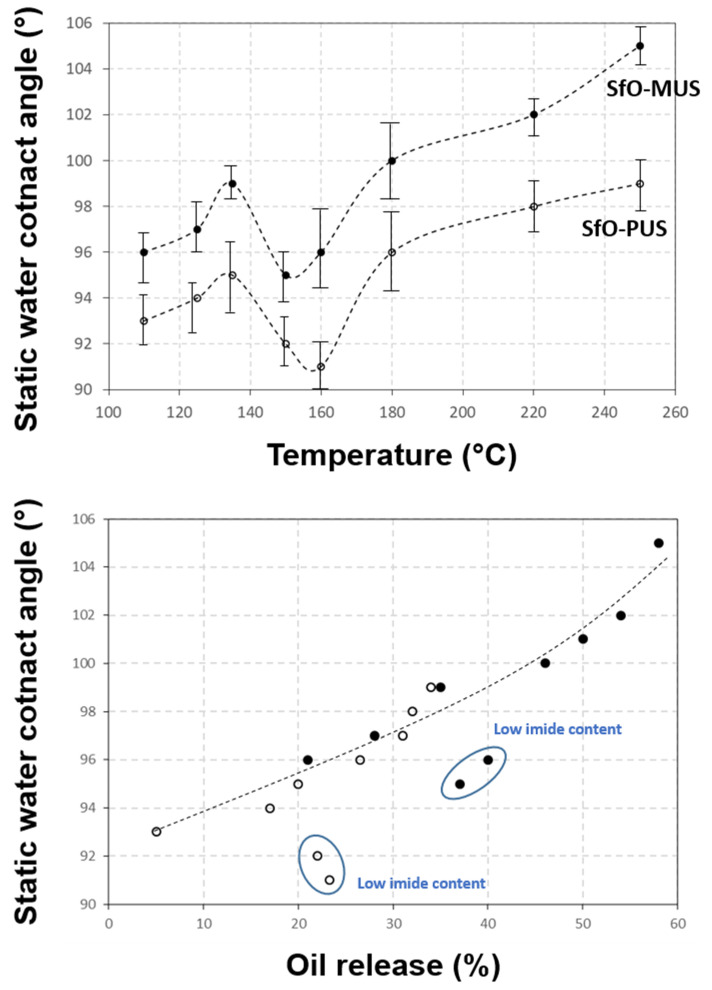
Hydrophobicity of SMI/SfO-PUS and SMI/SfO-MUS coatings on paper substrates after thermal oil release at different temperatures: (**a**) static water contact angles, (**b**) relation between water contact angle and oil release.

The variations in surface hydrophobicity of the coatings in parallel with the release of oil and/or increase in imide content are given in Figure 7. The indication of the statistical variation on static water contact angles is acceptable in view of the intrinsic heterogeneity of paper samples. The contact angles for both types of coated papers are in the hydrophobic range and show some parallel trends depending on the temperature (Figure 7a). The values for SMI/SfO-MUS coatings are significantly higher than SMI/SfO-PUS coatings over the full temperature range, and are in accordance with the higher amount of free oil at the surface. Therefore, the relative amount of free oil predominantly determines the degree of hydrophobicity. On the other hand, the static water contact angles increase towards a maximum at 135 °C, which can be related to the high imide content at this temperature. Indeed, the imidization reaction of a remaining fraction of ammonolyzed maleic anhydride evidently reduces the hydrophilic properties of the ammonolyzed maleic anhydride due to the higher tendency for water interaction with ammonolyzed and carboxylated groups compared to imide groups. As the imide content slightly reduces in the transition region at 135 to 150 °C, a parallel decreasing trend in the water contact angle is thus noticed. It is clear that both a lower imide content as well as a lower content of free oil is promoting the hydrophilic properties at 135 to 150 °C. In particular, the gradual release of oil predominantly favors surface hydrophobicity, with good overlap in free oil content and water contact angle for both coating types (Figure 7b).

**Figure 8:**
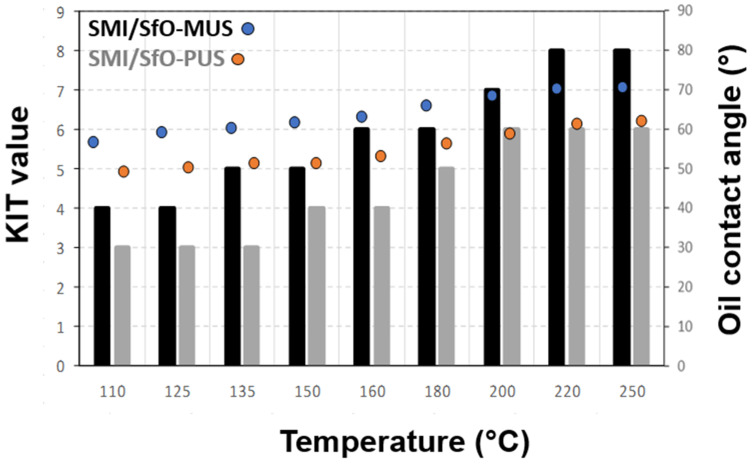
Oil resistance of SMI/SfO-PUS and SMI/SfO-MUS coatings on paper substrates as represented by the KIT values (bars) and contact angles for castor oil (dots).

Figure 8 includes the castor oil contact angles and KIT numbers. The oil contact angle for the uncoated paper is about 35°, but is not stable due to rapid absorption in the substrate. The values become more stable and increase after coating. It is interesting to notice that the oil contact angles further increase with progressive release of the oil in parallel with the amount of exposed oil at the surface. As such, the presence of free oil at the surface seems to form a natural barrier layer against oil penetration in the paper. In addition, the values from KIT testing increase for both coatings after thermal oil release, presumably as the coating layer becomes more homogeneously covered with oil.

### 3.3. Chemical Surface Mapping by Micro-Raman Spectroscopy

The chemical surface composition of coated papers is imaged by micro-Raman spectroscopy mapping, representing either band intensity ratios, single band intensities, or chemimaps. The normalization of spectra for single band intensities and chemimaps was performed relative to the integrated area of the cellulose band region 1170 to 1050 cm^−1^. The normalization of spectra for band intensity ratios was done according to the selected bands. As such, the lateral distribution of specific chemical moieties at the surface can be visualized in parallel with the evolution in intensities and surface coverage after heating at different temperatures.

**Figure 9:**
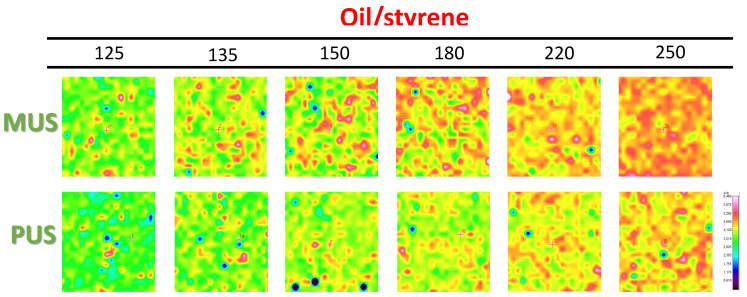
Raman spectroscopy surface maps of SMI/SfO-MUS and SMI/SfO-PUS coatings (1 × 1 mm^2^), representing band intensity ratio of oil/styrene (same intensity scale for all maps).

The exposure of oil at the coating surface after heating at different temperatures can be followed from the Raman maps with band intensity ratio of 1748 cm^−1^ to 1600 cm^−1^ (oil/styrene) in Figure 9. The oil and styrene moieties both refer to the coating and are suitable to illustrate the distribution of oil moieties within the coating. When using the same intensity scale for all surface maps, the presence of oil can be noticed from a higher intensity in red color. At low temperatures, the oil seems to be localized in rather discrete spots corresponding to zones with agglomerated nanoparticle deposits, while a more continuous film of oil progressively builds over the surface during thermal release. The variations in oil intensity of SMI/SfO-MUS coatings suggest that the oil content at the surface is highest and continuously increases in intensity with higher temperatures. The oil concentrations of SMI/SfO-PUS coatings are lower and remain relatively constant in the temperature range 135–180 °C, while only increasing at temperatures above 180 °C. The presentation of oil according to Raman mapping confirms previous oil release profiles that were determined from single-point measurements.

**Figure 10:**
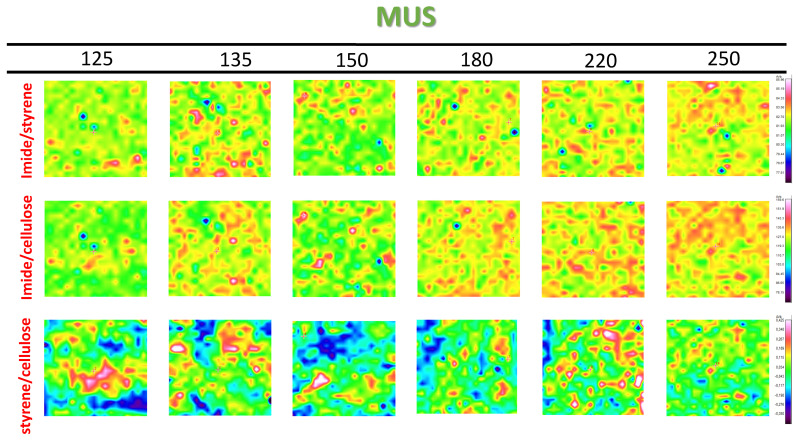
Raman spectroscopy surface maps of SMI/SfO-MUS coatings (1 × 1 mm^2^), representing band intensity ratio of imide/styrene, imide/cellulose, styrene/cellulose (same intensity scale per row).

**Figure 11:**
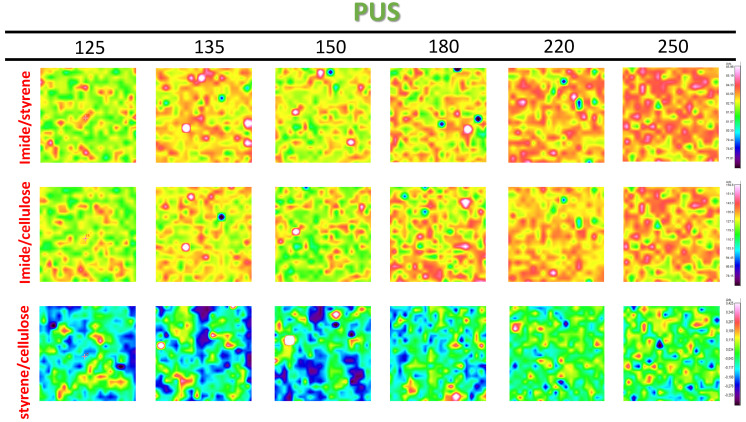
Raman spectroscopy surface maps of SMI/SfO-PUS coatings (1 × 1 mm^2^), representing band intensity ratio of imide/styrene, imide/cellulose, styrene/cellulose (same intensity scale per row).

The intensity and distribution of organic moieties in the coating itself and relative to the paper substrate is followed from Raman maps with band intensity ratios for SMI/SfO-MUS coatings in Figure 10 and SMI/SfO-PUS coatings in Figure 11. The band intensity ratios of 1785 cm^−1^ to 1602 cm^−1^ (oil/styrene) represent the variations in imide content in the coating itself against the inert styrene moieties. The imide intensities are indeed higher for SMI/SfO-PUS compared to SMI/SfO-MUS and have a locally higher intensity after heating at 135 °C, in agreement with previous single-point measurements. The lateral distribution of the imide phase is initially homogeneously concentrated in local points and progressively spreads over a lager surface area after heating at temperatures of 220 to 250 °C. It can be confirmed that the coatings with higher amounts of free oil correspond to the presence of lower imide content, as the organic imide phase acts as a rigid phase that binds the oil and prevents release. When taking the paper substrate as a reference, the band intensity ratios of 1780 cm^−1^ to 1095 cm^−1^ (imide/cellulose) and 1600 cm^−1^ to 1095 cm^−1^ (styrene/cellulose) illustrate the coating coverage over the substrate. The locations with high imide concentrations indeed coincide with the locations with high styrene concentrations. Both surface maps are an indication of locations where high amount of coating deposits occur. The locations with a high intensity of imide relative to the substrate (imide/cellulose) also largely correspond with locations where high imide intensity occurs in the coating itself (imide/styrene). While the intensity of imide/cellulose ratio over the substrate varies in intensity at higher temperature as before, the intensity of the styrene/cellulose ratio remains almost constant. At the higher temperatures, however, the styrene/cellulose ratio becomes more even on the surface due to the more homogeneous coverage of the coating above the glass transition temperature.

**Figure 12:**
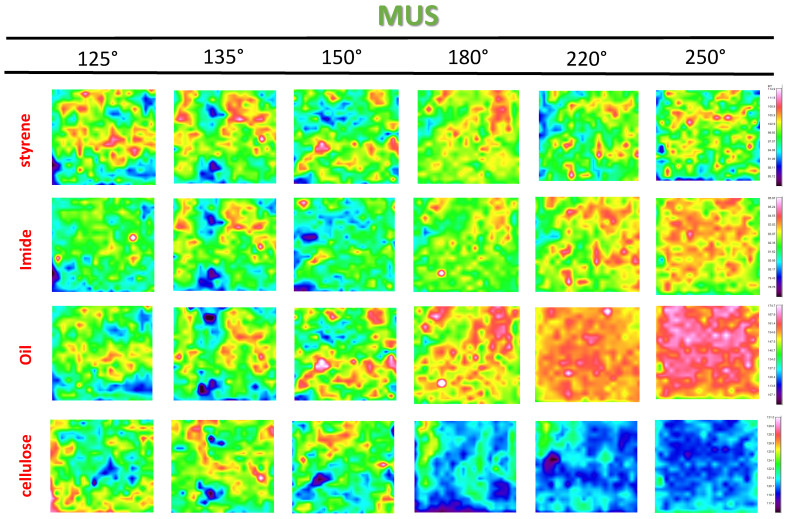
Raman spectroscopy surface maps of SMI/SfO-MUS coatings (1 × 1 mm^2^), representing single band numbers for styrene, imide, oil, and cellulose (same intensity scale per row).

The surface coverage for different components of the coating can be illustrated from the Raman maps with single band intensities, as shown for SMI/SfO-MUS coatings at different temperatures in Figure 12. The organic moieties (styrene, imide) cover comparable surface locations, with an almost constant intensity of the styrene band (1600 cm^−1^) and an increasing intensity of the imide band (1780 cm^−1^) towards a local maximum at 135 °C. The surface locations corresponding to higher intensity of the cellulose band (1095 cm^−1^) are complementary to the organic coating deposits, as the coating coverage varies depending on the porosity of the base substrate. The surface locations with thin coating deposits agree with the locations where the cellulose fibers become more present at the surface. Mainly the intensity of the cellulose band reduces at high temperatures as the paper substrate becomes more homogeneously covered with the coating. The surface areas presenting a high intensity of the oil band (1748 cm^−1^) overlap with the surface areas of organic coating deposits; however, it is confirmed that the release of oil starts to cover the entire surface at higher temperatures above *T_g_* in parallel with the viscous flow of the coating.

**Figure 13:**
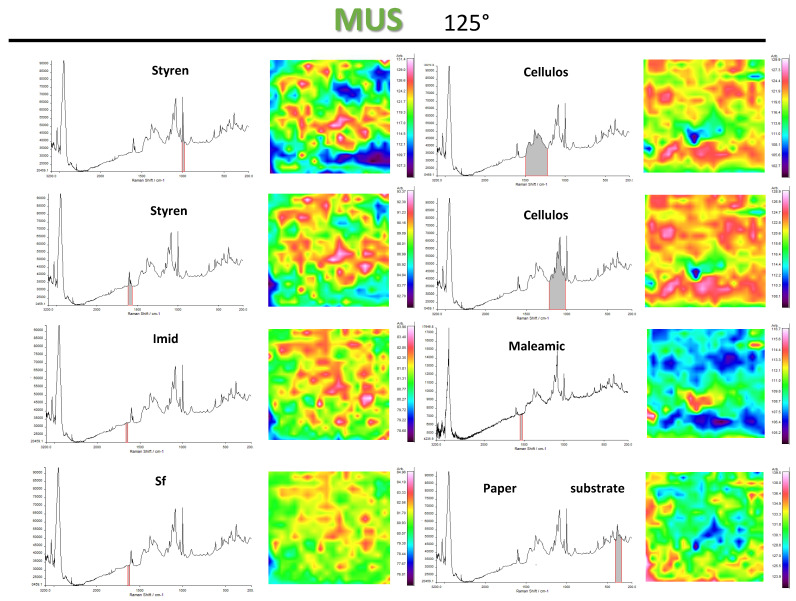
Raman spectroscopy surface maps of SMI/SfO-MUS coatings at 125 °C (1 × 1 mm^2^), representing chemimaps for different spectral regions.

The detailed information on coating homogeneity (e.g., surface defects), and surface interactions in particular, can be obtained from Raman chemimaps in Figure 13, presented for SMI/SfO-MUS coatings at 125 °C. The chemimaps for styrene cover the same areas, but with a higher intensity of the 1600 to 1583 cm^−1^ band region (i.e., aromatic C=C stretching) versus the 1032 to 1000 cm^−1^ band region (C–H bending): although the coatings have a constant styrene content, the relative variations in both intensities can eventually be attributed to the differences in styrene orientation relative to the hydrophobic oil phase.

The surface coverage of the imide band (1780 cm^−1^) and oil band (1657 cm^−1^) overlaps at the same locations and is complementary to the locations covered by the cellulose band region (1170 to 1050 cm^−1^). The different intensities of both cellulose regions at 1500 to 1200 cm^−1^ (bending of H–C–C, H–C–O, H–C–H) and 1170 to 1050 cm^−1^ (stretching of H–O–C and C–O–C) relate to the different assignments of these bands, where the higher intensity of H–O–C stretching might be introduced through the presence of hydrogen bond interactions between the nanoparticles and the cellulose, which is highest for the coatings with high imide content. The latter ensures good adhesion of the coating towards the paper substrate. The remaining Raman band at 1565 cm^−1^ corresponds to residual maleamic acid moieties of the ammonolyzed SMA precursor [53], which were not fully imidized after ammonolysis. They can indeed be recognized as coating defects at locations corresponding with low intensity of imide bands. It has been observed that the intensity of this band further decreases with increasing temperatures, as the imidization of ammonolyzed SMA proceeds at higher temperatures. The lower Raman band at 400 to 500 cm^−1^ relates to inorganic fillers in the paper (e.g., kaolinite) [54], and is complementary to the presence of cellulose fibers. The locations with the inorganic fillers seem to be less favorably covered with the organic coating layer, affirming a stronger interaction between the imide moieties and the cellulose fibers.

### 3.4. Quantification of Release Profiles from Micro-Raman Mapping

**Figure 14:**
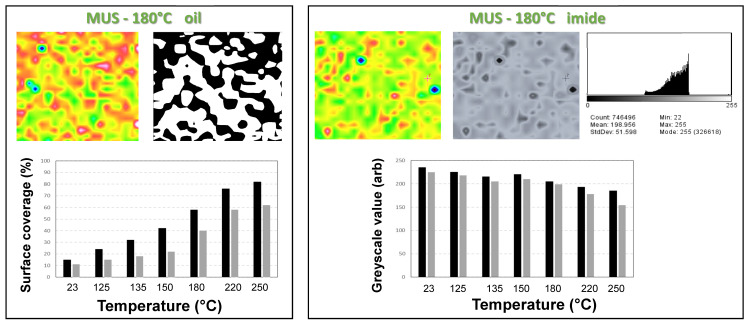
Quantification of chemical characteristics of SMI/SfO-PUS and SMI/SfO-MUS coatings after thermal oil release based on Raman surface mapping and image processing: (**a**) determination of oil release from surface area coverage, (**b**) determination of imide content from greyscale value.

The transformation of the original Raman maps into quantifiable data was done by image processing steps that are illustrated in Figure 14. The determination of the surface area coverage (Figure 14a) was done for quantifying the oil release, and the determination on the greyscale value (Figure 14b) allowed for following the imide content.

The determination of the surface area coverage (%) is illustrated for a particular case of a SMI/SfO-MUS coating at 180 °C (Figure 14a), selected from the Raman map with band intensity ratio oil/styrene (see previous Figure 8). The image processing illustrates good correspondence in detecting the borders of surface features related to oil deposits and automatic calculation of the black area. When plotting the surface area coverage (%) corresponding to the oil deposits as a function of heating temperature, the trends for both SMI/SfO-MUS and SMI/SfO-PUS coatings describe the release profile in parallel with the earlier release profiles calculated from the single-point measurements. The SMI/SfO-MUS coating shows a large and progressive release over the temperature range, while the release for the SMI/SfO-PUS coating is restricted below 180 °C, as explained before. As such, a good quantification for oil release from the paper coatings based on chemical surface mapping has been established.

The determination of the greyscale value is also monitored for the same SMI/SfO-MUS coating at 180 °C (Figure 14b), selected from the Raman map with band intensity ratio imide/styrene (see previous Figure 9). The histogram after conversion of the Raman map into a greyscale image allows one to read an average value between 0 (black) and 255 (white) as an arbitrary number. The evolution of the greyscale values for different coatings corresponds to the changes in imide content at the surface as a function of temperature. As the imide is indeed rather related to the color intensity of the original Raman map, the black color (i.e., lower greyscale value) corresponds to a higher intensity and higher imide content. The trends correspond well to the previous calculations of imide content according to the single-point measurements, with a higher imide content for SMI/SfO-PUS than SMI/SfO-MUS and a local maximum at 135 °C. As such, the variations in imide content can be estimated from the surface mapping and quantified after calibration with previous single-point measurements.

## 4. Conclusions

The thermal release of oil from a nanoparticle coating on paper shows strong sensitivity of the release profiles towards the composition of encapsulated oil. While using two sunflower oils with different content of poly- and monosaturated fatty acids, different release profiles of oil from the nanoparticle coatings were observed. The amount of free oil depends on the degree of saturation of the oil and is the highest for the more saturated sunflower oil that mainly contains mono-unsaturated fatty acids. These properties are related to different reactivity of both oil types during imidization of the nanoparticles. As the poly-unsaturated oils are more reactive, they are better encapsulated by the polymer phase of the nanoparticles. Consequently, the oil release is the highest for the mono-unsaturated sunflower oil, and the oil release is retarded for the poly-unsaturated sunflower oil. In parallel, the hydrophobicity and oil resistance of the coated papers increases in parallel with the trends in oil release for both coating types.

The thermal release of oil can be successfully quantified and visualized by micro-Raman spectroscopy and chemical surface mapping. Depending on the temperature, the presentation of oil and reactivity of the imide phase within the coating is distinguished for both encapsulated oil types. The coating coverage relative to the paper substrate is complementary to the occupation of the cellulose bands and further homogenizes at the higher temperatures. In particular, data obtained from image processing can be used for the quantification of the oil content (surface area coverage), or imide content (greyscale value).

In conclusion, the features of the present system can be very well controlled and are applicable for tuning required surface properties of papers for packaging applications. Besides the improvements in hydrophobicity and barrier properties, the paper coating may be further adapted for delivery of oil or oil-soluble compounds, fragrances, preservatives, antimicrobial agents, and so on in future applications for active packaging. Although good physico-chemical adhesion between the nanoparticles and the paper substrate is demonstrated, the final implementation for commercial applications would require the reduction of migration of free styrene, which can be controlled by a post-polymerization step or addition of scavengers. Besides the use in packaging papers, the application in specialty papers with release at high temperatures is of interest. The present coatings applied as aqueous dispersion coatings provide potentially good repulpability, as shown in preliminary experiments, which is an issue for future research.

## Data Availability

The data presented in this study are available on request from the corresponding author.

## Sample Availability

Samples of the coating materials are available from the author.
